# The Beginning of Senior Career in Team Sport Is Affected by Relative Age Effect

**DOI:** 10.3389/fpsyg.2019.01465

**Published:** 2019-06-26

**Authors:** Corrado Lupo, Gennaro Boccia, Alexandru Nicolae Ungureanu, Riccardo Frati, Roberto Marocco, Paolo Riccardo Brustio

**Affiliations:** ^1^NeuroMuscularFunction Research Group, School of Exercise & Sport Sciences, Department of Medical Sciences, University of Turin, Turin, Italy; ^2^School of Exercise & Sport Sciences, SUISM, University of Turin, Turin, Italy

**Keywords:** talent identification, biological age, team sports, physical maturation, RAE

## Abstract

Many previous studies in national team sports did not report evidence about relative age effect (RAE) in senior categories. This study aimed for the first time to determine if the RAE may specifically affect the early, but not the late, phase of senior career in elite team sports. A total of 3,319 birthdates (basketball: *n* = 642; rugby: *n* = 572; soccer: *n* = 1318; volleyball: *n* = 337; water polo: *n* = 450) of elite senior players were analyzed. Senior players with an age lower or equal to the 25° percentile of age were considered as *early phase* players while the others as *late phase* players. Separate Poisson regression models were applied to investigate the RAE in each sport (overall, and for *early phase* and *late phase* subgroups). Considering the overall sample, players born close to the beginning of the year were 1.57, 1.34, 2.69, 1.48, and 1.45 times more likely to reach first and second Italian division of basketball, rugby, soccer, volleyball, and water polo respectively, than those born in the last part of the year. RAE was present in all *early phase* subgroups. Differently, in the *late phase* subgroups the RAE was present only in soccer. Data highlighted a bias in the selection of senior teams, which may limit the chance to identify talented players born late in the second part of the year. Italian sport federations should promote the talent development of relatively younger players by equally promoting the joining of young players to senior teams.

## Introduction

In sport context, birthdates are usually chosen to gather sport categories of adolescents and young athletes. Literature reported that in youth teams’ relatively older players (i.e., athletes born close to cut-off date of selection) and relatively younger players (i.e., athletes born far away to cut-off date of selection) may present large differences in physical and psychological maturation (Cobley et al., 2009; Lovell et al., 2015). As consequence, relatively older players are advantaged in sport performance if compared with relatively younger players (Cobley et al., 2009) and have more probabilities to be selected by coaches and talent scouts (Lovell et al., 2015; Furley and Memmert, 2016; Sarmento et al., 2018). This leads to an over-representation of athletes born close to selection date. This phenomenon is recognized with the name of Relative Age Effect (RAE) (Barnsley et al., 1985; Musch and Grondin, 2001; Boccia et al., 2017).

The RAE has been successively observed in several individual (Cobley et al., 2018; Kearney et al., 2018; Brustio et al., 2019) and team sports. In the latter sport area, this phenomenon has been especially investigated in soccer (Fumarco and Rossi, 2015; Gonzalez-Villora et al., 2015; Sierra-Diaz et al., 2017; Brustio et al., 2018; Rađa et al., 2018), basketball (Arrieta et al., 2016; Rubajczyk et al., 2017; Steingrover et al., 2017; Ibanez et al., 2018), and rugby union (Till et al., 2010; McCarthy et al., 2016; Jones et al., 2018), but also in other team sports such as volleyball (Campos et al., 2016) and water polo (Barrenetxea-Garcia et al., 2018). Despite the pervasiveness of RAE in youth sport teams, controversial results were found at senior professional level (Delorme et al., 2009; Romann et al., 2018). For example, large RAE was found in soccer (Gonzalez-Villora et al., 2015; Brustio et al., 2018), while small or negligible RAE was observed in other sport teams such rugby league (Delorme et al., 2009; Till et al., 2010; Jones et al., 2018), basketball (Delorme et al., 2009; Werneck et al., 2016; López de Subijana and Lorenzo, 2018), volleyball (Campos et al., 2016) and water polo (Barrenetxea-Garcia et al., 2018). A discrepancy in RAE at senior professional level may also emerge in relation to the countrywide. For example, in a nationwide analysis of most popular sports in Switzerland (Romann et al., 2018), despite authors found an overall RAE in male athletes, RAE associated to single sports reported substantial differences. Similarly, Delorme et al. (2009) showed in French male professional players a different pattern in RAE according to the sport teams considered. Specifically, a significant RAE was observed only in ice-hockey while only a trend was detected in handball and rugby union but not in basketball, soccer or volleyball. Interesting, a different trend was observed in female professional players, where RAE was found to be small or not present at all (for an extensive review and meta-analysis see Smith et al., 2018). Indeed, the social context, the level of competition, the popularity, and number of active participants may affect the presence of RAE (Musch and Grondin, 2001). The paucity of studies focused on RAE of different team sports in the same countrywide is evident, and further studies are strongly needed.

New evidences underlined a reversal RAE at senior professional level, where relative younger players may have the greatest potentiality for a later success (McCarthy et al., 2016; Till et al., 2016). In other words, it is possible that relative younger players may have the chance to be identified and selected at senior professional level at late stage of their career. Accordingly, as previously suggested, it is possible that RAE magnitude may differ in the early and late phase of professional senior career (Brustio et al., 2018). Indeed, it is likely to hypothesize that only the first years of senior career may be affected by the RAE. This would mean that the relatively older athletes (i.e., those born close to the selection date) may be advantaged in entering the senior teams. Despite this sensible hypothesis, previous studies, focusing on RAE at senior level, investigated the senior teams without differentiating between the athletes in the first years of senior career and the others (Delorme et al., 2009; Till et al., 2010; Werneck et al., 2016; Jones et al., 2018; López de Subijana and Lorenzo, 2018).

Thus, the aim of this study was to quantify the prevalence and magnitude of RAE in male Italian context at professional senior level (i.e., first and second division), focusing on basketball, rugby (i.e., rugby union, 15 players each team), soccer, volleyball, and water polo, which are the most popular team sports in Italy^1^. In addition, RAE was separately quantified for players competing in the early (*early phase* players) and later (*late phase* players) stage of their senior career in order to determine if the RAE in senior team may be influenced over time.

## Materials and Methods

### Design

Birthdates of 3,319 male athletes were collected from the first and second Italian division of basketball, rugby, soccer, volleyball and water polo for the 2017–2018 seasons. Date of birth was obtained from different sources such as TransferMarkt database for soccer players and web sites of clubs for the other sports. Data collection has been performed at the end of the season, when the teams’ rosters are consolidated after eventual players moving among different clubs. All players included in the rosters of senior teams were considered in this analysis regardless of the age.

### Procedure

A total of 642 (mean age = 25.6 ± 5.6 years), 572 (mean age = 24.2 ± 4.5 years), 1318 (mean age = 25.0 ± 5.0 years), 337 (mean age = 25.6 ± 5.5 years), and 450 (mean age = 25.6 ± 6.3 years) male elite players were analyzed in basketball, rugby, soccer, volleyball, and water polo, respectively. The 18, 7, 9 4, and 24 % of the subjects in basketball, rugby, soccer, volleyball, and water polo, respectively aged less than 18 years. Players’ names were removed from the data set. Since our data are based on anonymous resources, no informed consent was requested. This study was approved by the local ethics committee of the University of Turin (Italy) and conducted in accordance with the declaration of Helsinki.

### Statistical Analyses

To explore the possible differences in RAE between *early phase* and *late phase* players, the whole sample of each sport was split in two subgroups on the basis of their age. Those senior players with an age lower or equal to the 25° percentile of age of the considered sport (i.e., the first quartile of players in terms of age) were considered as *early phase* players; the rest of sample was considered as *late phase* players. It is likely that *early phase* players (i.e., the younger players in the senior sample) were those in their first years of their adult career. In absence of the actual year of entering the senior team, the age of the player has been considered as the most sensible index to determine if a player is in the early or late phase of his adult career.

In Italy, the youth categories are based on calendar years, for which all young players born from 1st January to 31st December of a calendar year are grouped together. Thus, the birth week (W_B_) and the time of birth (T_B_) of each player were computed according to previous studies (Brustio et al., 2018, 2019; Doyle and Bottomley, 2018, 2019). W_B_ indicates the week of the year of player’s birth while T_B_ indicates how far from the beginning of the year a player was born (range score between 0 and 1).

In line with recent studies on RAE (Brustio et al., 2018, 2019; Doyle and Bottomley, 2018, 2019; Rađa et al., 2018), a separate Poisson regression was applied to investigate the RAE in each team sport following the formula *y* = e^(b0+b1x)^. Specifically, *y* represents the frequency of birth in a given week while *x* represents the T_B_. Moreover, the Index of Discrimination (ID), which provided the relative odds of being selected for a player born in the first versus last week of the competition year (Doyle and Bottomley, 2018, 2019), was calculated as e^-b1^. Finally, likelihood ratio *R*^2^ was computed (Cohen et al., 2013). All data were analyzed in MATLAB R2017b (Mathworks, Natick, MA, United States).

## Results

**FIGURE 1:**
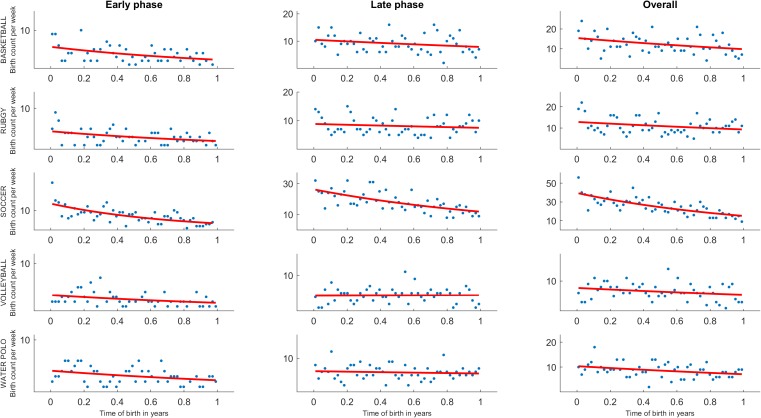
Scatter-plots of birthdates reported for each team sport (i.e., basketball, rugby, soccer, volleyball, and water polo) in relation to the entire samples, and to the *early phase* and *late phase* career subgroups. The red line represents the best fit of the Poisson regression.

Figure 1 reports the scatter-plots of relative birth frequency by week, the red line in the Figure 1 represents the best fit of the Poisson regression modeling the frequency of birth in a given week the T_B_. Table 1 shows the descriptive statistic of W_B_ and T_B_, and the output of Poisson regression for each subgroup and sport. The Table 1 shows that the players of each considered sport were born at beginning of the year (mean T_B_ range: 0.42–0.48). Moreover, players born close to the beginning of the year (i.e., in the first week of the year) were 1.57, 1.34, 2.69, 1.48, and 1.45 times more likely to reach first and second Italian division of basketball, rugby, soccer, volleyball, and water polo than those born in the last part (i.e., last week) of the year (see ID values).

**Table 1:** Poisson regression results in basketball, rugby, soccer, volleyball, and water polo.

		Basketball	Rugby	Soccer	Volleyball	Water polo
Overall	N	642	572	1318	337	450
	W_B_	25 ± 15	25 ± 16	22 ± 14	25 ± 14	25 ± 15
	T_B_	0.46 ± 0.29	0.48 ± 0.3	0.42 ± 0.28	0.47 ± 0.27	0.45 ± 0.29
	b_0_	2.732	2.541	3.687	2.058	2.337
	b_1_	-0.454	-0.294	-1.000	-0.392	-0.370
	**ID**	**1.57**	**1.34**	**2.69**	**1.48**	**1.45**
	*R*^2^	0.12	0.07	0.6	0.07	0.11
	*p* value	<0.0001	0.04	<0.0001	0.04	0.02
Early phase	N	168	149	376	115	147
	W_B_	23 ± 15	24 ± 16	21 ± 14	23 ± 15	23 ± 15
	T_B_	0.42 ± 0.28	0.44 ± 0.30	0.39 ± 0.27	0.42 ± 0.28	0.43 ± 0.28
	b_0_	1.714	1.457	2.581	1.243	1.415
	b_1_	-0.866	-0.729	-1.333	-0.716	-0.611
	**ID**	**2.38**	**2.07**	**3.79**	**2.05**	**1.84**
	*R*^2^	0.19	0.14	0.47	0.16	0.12
	*p* value	<0.0001	0.01	<0.0001	0.03	0.04
Late phase	N	474	423	942	222	303
	W_B_	25 ± 15	26 ± 16	23 ± 14	26 ± 14	26 ± 15
	T_B_	0.48 ± 0.29	0.49 ± 0.3	0.43 ± 0.28	0.49 ± 0.27	0.49 ± 0.29
	b_0_	2.351	2.171	3.283	1.472	1.820
	b_1_	-0.288	-0.152	-0.829	0.037	-0.145
	**ID**	**1.33**	**1.16**	**2.29**	**0.96**	**1.12**
	*R*^2^	0.06	0.02	0.47	0	0.01
	*p* value	0.07	0.37	<0.0001	0.87	0.56

*W_B_, week in which players born; T_B_, time of birth; ID, Index of Discrimination*.

The cut-off ages for discriminating *early phase* and *late phase* subgroups were: 19 years, for basketball and water polo; 21 years, for rugby and soccer; and 22 years, for volleyball. According to these scenarios, the results suggested that the amount of RAE was greater in the *early phase* than *late phase* subgroup of players in each sport. In fact, the Poisson regressions were significant for *early phase* players in basketball (*p* < 0.0001; *R*^2^ = 0.19), rugby (*p* = 0.01; *R*^2^ = 0.14), soccer (*p* < 0.0001; *R*^2^ = 0.47), volleyball (*p* < 0.03; *R*^2^ = 0.16), and water polo (*p* < 0.04; *R*^2^ = 0.12). Differently, Poisson regression was significant for *late phase* subgroups, only in soccer (*p* < 0.0001; *R*^2^ = 0.47), but not in the other sports. The T_B_ indexes in all sports were lower in *early phase* compared to *late phase* subgroups (see Figure 1). In addition, coherently to the above-mentioned results, IDs indexes in all sports were greater in *early phase* compared to *late phase* subgroups (see Table 1).

## Discussion

The aim of this study was to examine the prevalence and magnitude of RAE at senior professional level in Italian team sports, discriminating male players in relation to *early* or *late phase* of career. For this purpose, the birthdates distribution of basketball, rugby, soccer, volleyball, and water polo players competing in the first and second Italian Championships was recorded. The main finding consisted of a large over-representation of players born close to the beginning of the calendar year in early, but not in late, phase of senior career. Soccer was an exception, showing that even *late phase* players presented evidence of RAE, despite smaller than *early phase*.

Considering the overall sample, evidence of RAE was found in each considered team sport. To quantify the RAE, we adopted the ID which consists in the relative odds of being selected for an athlete born in the first versus the last week of the calendar year (Doyle and Bottomley, 2018, 2019). In this study, the IDs ranged from 1.34 to 2.69 underlined the presence of RAE in all considered team sports. Thus, the present results showed a different pattern in senior Italian context compared to International ones, where RAE was small or negligible as observed in basketball (Delorme et al., 2009; Werneck et al., 2016; López de Subijana and Lorenzo, 2018), rugby league (Till et al., 2010), volleyball (Campos et al., 2016) and water polo at senior level (Barrenetxea-Garcia et al., 2018). On the other hand, it possible that the proportion of players with less than 18 years (overall mean = 12%) in analyzed teams may partially influence the results contradicting previous results in senior teams and in accordance with other studies which describe RAE in European National Teams (Arrieta et al., 2016). However, as outlined before, controversial results may arise in relation to the countrywide, because the social context, the level of competitiveness, the popularity, and number of active participants affect the magnitude of RAE (Musch and Grondin, 2001; Cobley et al., 2009; Wattie et al., 2015). Thus, this finding should be considered specific to Italian context and cannot be generalized to other countries. Some speculations could be provided to more deeply interpret the results of the present study. The team sports of our study may be considered as highly physical demanding sports, where strength and power represent a substantial role. Since physical maturation is related to muscular strength, endurance, and speed, and is important to obtain successful performances (Musch and Grondin, 2001; Cobley et al., 2009), relatively older players could be more favored than younger counterparts. Moreover, soccer, basket, rugby, and water polo are invasion sports and characterized by physical contacts, where higher muscular strength and body dimensions are directly favorable. Differently, despite volleyball is not an invasion sport, greater body dimension and physical capabilities can be favorable as well. This can explain the fact that also volleyball presented RAE values in line with the other sports, even if it is not an invasion sport. Beyond the physical aspect, the influence of social agents such as parents, coaches or the athletes themselves, especially at the beginning of the careers, may amplify RAE (e.g., best coaches, facilities, equipment, higher self-expectations) (Hancock et al., 2013). Furthermore, also environmental factors, such as birthplace, may positively impact on athletes’ development and increase the opportunity to obtain success in sport (Côté et al., 2006).

Soccer is the sport more affected by RAE (Table 1). Players born near the cut-off date of selection are nearly three times more likely to play in the first or second division. The larger RAE in soccer may be related to the fact that soccer is the most popular sport in Italy. In fact, the high professionalism of the first and second division can be associated to a more severe player selection in comparison with other team sports. Moreover, the greater attractiveness of soccer compared to other sports in term of media presence and higher funding (Romann et al., 2018) might explain the larger RAE in soccer. In other words, we may speculate that the relative inferior popularity and number of active participants of basketball, rugby, volleyball, and water polo in Italy, and consequent more opportunities to be selected in elite teams may have minimized the RAE. As consequence, even though soccer can benefit from a huge number of players, its talent selection seems to risk more than other team sports to lose valuable elite players only because of a late physical maturation occurring during the youth phase of career. Therefore, it could be hypothesized a paradoxical phenomenon, where an effective and trivial talent selection is developed in minor team sports and soccer, respectively.

The most interesting result of this study was that in basketball, rugby, volleyball, and water polo, the RAE was present only in *early phase* subgroup (IDs ranged = 1.84–2.38) but not in the *late phase* subgroup (IDs ranged = 0.96–1.33). This finding highlights that, in these sports, relatively younger players may still suffer the initial disadvantage undergone in youth career (Cobley et al., 2009) during their *early phase* of adult career (McCarthy et al., 2016; Till et al., 2016). In soccer, the results presented the same trend but with even more pronounced RAE. Indeed, the ID was extraordinarily large in *early phase* subgroup (ID = 3.8) and lower, but still large, in *late phase* subgroup (ID = 2.3). Taken together, the present findings show that the relatively older soccer players (i.e., those born close the beginning of the year) are facilitated in entering the senior professional teams. Moving from youth to senior team is a delicate passage in players’ careers. However, it seems that the relatively older players have early chances to join the senior teams compared to the relatively younger players. Anyway, for relatively younger players, further opportunities to play with senior team should be offered to optimize the process of talent development. However, it is known that the excessive research of immediate successes in youth categories could prevent the promotion of long-term talent development, leading coaches to select youth players with more mature physical characteristics (Lovell et al., 2015; Furley and Memmert, 2016; Sarmento et al., 2018).

This study presents some limitations. The study considered the whole roster of the selected teams. Hence, it included also foreign players as well as players with less than 18 years. These aspects could potentially influence the observed RAE and the cut-off age used to categorize *early phase* and *late phase* players. Moreover, the present study did not investigate the possible causal factors of RAE, such individual performance during a season (e.g., total minutes and number of the match played) or individual characteristics (e.g., physical fitness performance, physical maturation, and anthropometrical measures) which may better describe the phenomenon. Additionally, the present study focused on male competitions only. Due to the possible small effect of RAE in female sport contexts (Smith et al., 2018), future studies are needed to investigate this phenomenon in the *early* and *late* phase of female players’ career. Finally, the present study considered only one year of completion (e.g., 2017–2018 season). Furthermore, studies are needed to investigate the RAE considering the starting age of the players in elite teams as well as the phenomenon longitudinally.

## Conclusion

At senior professional level, a large over-representation of players born close to the beginning of the year is evident in all popular Italian team sports. However, this trend significantly emerged for the *early phase* of players’ career, whereas it was weaker for the *late phase*, which was significant only in soccer. Therefore, these data suggested that relatively older players have more chances to join senior teams especially at the beginning of their adult career.

To limit the negative RAE consequence, Italian sport federations should provide different solutions to advantage all athletes with different time points of development (Hurley et al., 2001; Romann and Cobley, 2015; Mann and van Ginneken, 2017; Haycraft et al., 2018). Since results of present study showed that the RAE is more evident in the early phase of players’ career, practitioners should try to find solutions to support athletes in the transition from youth to senior teams. Taking in mind that even relatively younger players can reach top-level senior competition, team sports coaches should consider the later development trajectories of youth athletes. Thus, they should promote the talent development of the relatively younger players by equally promoting the joining of young players to senior teams.

## Data Availability

The datasets generated for this study are available on request to the corresponding author.

## Author Contributions

CL, GB, and PRB conceptualized and supervised the study and wrote the original draft of the manuscript. All authors investigated the study, wrote, reviewed, and edited the manuscript. PRB carried out the formal analysis. CL acquired the funding.

## Conflict of Interest Statement

The authors declare that the research was conducted in the absence of any commercial or financial relationships that could be construed as a potential conflict of interest.

## Footnotes

1 https://www.coni.it
